# Combination Treatment with Liposomal Doxorubicin and Inductive Moderate Hyperthermia for Sarcoma Saos-2 Cells

**DOI:** 10.3390/ph17010133

**Published:** 2024-01-19

**Authors:** Valerii E. Orel, Anatoliy G. Diedkov, Vasyl V. Ostafiichuk, Oleksandra O. Lykhova, Denys L. Kolesnyk, Valerii B. Orel, Olga Yo. Dasyukevich, Oleksandr Yu. Rykhalskyi, Serhii A. Diedkov, Anna B. Prosvietova

**Affiliations:** 1 National Cancer Institute, 33/43 Zdanovska Str., 03022 Kyiv, Ukraine; 2 National Technical University of Ukraine “Igor Sikorsky Kyiv Polytechnic Institute”, 16/2 Yangel Str., 03056 Kyiv, Ukraine; 3 R.E. Kavetsky Institute of Experimental Pathology, Oncology and Radiobiology, 45 Vasylkivska Str., 03022 Kyiv, Ukraine

**Keywords:** osteosarcoma, Saos-2 cells, liposomal doxorubicin, inductive moderate hyperthermia, combination treatment, antitumor effect, reactive oxygen species, early apoptosis, Bax protein, tumor cell heterogeneity

## Abstract

Despite efforts in osteosarcoma (OS) research, the role of inductive moderate hyperthermia (IMH) in delivering and enhancing the antitumor effect of liposomal doxorubicin formulations (LDOX) remains unresolved. This study investigated the effect of a combination treatment with LDOX and IMH on Saos-2 human OS cells. We compared cell viability using a trypan blue assay, apoptosis and reactive oxygen species (ROS) measured by flow cytometry and pro-apoptotic Bax protein expression examined by immunocytochemistry in response to IMH (42 MHz frequency, 15 W power for 30 min), LDOX (0.4 μg/mL), and LDOX plus IMH. The lower IC_50_ value of LDOX at 72 h indicated increased accumulation of the drug in the OS cells. LDOX plus IMH resulted in a 61% lower cell viability compared to no treatment. Moreover, IMH potentiated the LDOX action on the Saos-2 cells by promoting ROS production at temperatures of <42 °C. There was a 12% increase in cell populations undergoing early apoptosis with a less heterogeneous distribution of Bax after combination treatment compared to those treated with LDOX (*p* < 0.05). Therefore, we determined that IMH could enhance LDOX delivery and its antitumor effect via altered membrane permeabilization, ROS generation, and a lower level of visualized Bax heterogeneity in the Saos-2 cells, suggesting the potential translation of these findings into in vivo studies.

## 1. Introduction

While primary malignant bone tumors comprise less than 1% of all cancers, they pose a high cumulative burden, impairing physical function. These tumors exhibit an average standardized rate of 0.8–1.2 per 100,000 persons in males and 0.5–1.0 per 100,000 persons in females across all age groups globally. Osteosarcoma (OS) is the most common primary malignant bone tumor, accounting for 20–40% of all diagnoses. It has been suggested that OS originates from a mesenchymal cell lineage capable of producing an osteoid matrix. OS predominantly occurs in the metaphyses of long bones, namely, the distal femur, the proximal tibia, and the proximal humerus. Recent reports have highlighted an increase in OS incidence as high as three cases per one million people annually. The incidence of OS demonstrates a bimodal age distribution, with a significant peak during puberty that is linked to growth spurts and a less pronounced peak in older adults (>75 years) that is associated with Paget’s disease. Several factors contribute to an increased risk of OS development, including exposure to ionizing radiation, chemotherapy, farm chemicals, birth weight, and height. Although most OS cases are sporadic, approximately 70% of examined tumor specimens display chromosomal abnormalities. The majority of OS patients with localized disease are typically eligible for neoadjuvant therapy, surgery, and subsequent adjuvant treatment. However, OS survivors often experience long-term adverse effects and a poorer quality of life due to systemic chemotherapy [1,2,3,4,5].

Doxorubicin (DOX), an anthracycline antitumor antibiotic, serves as a first-line chemotherapy agent used in the treatment of bone sarcomas. The mechanisms by which DOX acts on OS cells involve the following: (1) the formation of free radicals, (2) DNA intercalation, and (3) the inhibition of topoisomerase-II (Top-IIA). Consideration should also be given to its potential in promoting host antitumor responses through immunogenic cell death. Stressed or dying OS cells, as a result of DOX action, were found to release damage-associated molecular pattern cytokines from degraded organelles. Such cytokines attract and stimulate immune cells in a microenvironment, leading to a more pronounced inhibition of tumor growth [6], and it immediately becomes apparent that there are toxicity issues associated with chemotherapy-induced damage to normal cells. For instance, DOX triggers apoptosis in cardiomyocytes by initiating reactive oxygen species (ROS) generation and iron accumulation [7].

With advancements in drug design, DOX is now administered in its free form or as a nanoparticle formulation. Various drug delivery platforms have been developed for DOX employing polymeric [8], protein [9], liposomal [10], and metallic nanoparticles [11]. While metal-based nanoparticles, particularly those with magnetic properties such as iron oxide (Fe_3_O_4_), offer a practical solution to targeted delivery and remotely controlled release of DOX under the influence of external electromagnetic fields, there are still some concerns regarding their long-term toxicity profiles [12]. Polymeric (polylactic acid, polyglycolic acid, or poly(lactic-co-glycolic acid)), protein (albumin), and liposomal nanoparticles demonstrate, on the other hand, high biocompatibility and biodegradability. These nanoparticle formulations possess different physicochemical properties that determine their behavior within the biological media. Liposomes, characterized by higher elasticity and reduced tendency for aggregation compared to other nanocarriers [13,14], can contribute to a more uniform distribution of the drug within a tumor. Furthermore, encapsulating DOX in an aqueous environment within a pegylated liposome protects the drug from degradation during circulation [15].

Liposomal DOX (LDOX) typically exhibits reduced toxicity, including cardiotoxicity, reproductive toxicity, and the development of secondary cancer, compared to its free form [16]. The latter is achieved through the passive targeting of LDOX to a tumor site, exploiting the enhanced permeability and retention effect (EPR). The larger fenestrae between endothelial cells (up to 800 nm in diameter) in tumor blood vessels allow for the extravasation of liposomes with diameters of ~100 nm. Moreover, the impaired lymphatic vessel networks in malignant tumors also contribute to the accumulation of LDOX. A growing body of evidence has confirmed that LDOX maintains its antitumor effect in OS models. LDOX has thus emerged as a potential candidate for targeted and controlled drug delivery, offering a slower release of DOX and prolonged systemic circulation. These properties of LDOX mitigate some adverse effects associated with the administration of high doses of its free form [17,18]. However, despite the more favorable tolerability of LDOX in cancer patients, in particular, those with advanced sarcoma, there have been no significant differences reported in the survival rates between treatment regimens using free and liposomal formulations of DOX [19,20,21,22,23].

There are a number of reasons why the passive targeting of LDOX encounters certain limitations as a therapeutic approach. Depending on tumor size, LDOX may fail to penetrate deep into a target because of its retention on the target’s surface, thereby leading to chemotherapy resistance. Another challenge is that LDOX distribution based on the EPR effect alone does not account for the heterogeneity of malignant tumors [24]. One approach to improve the delivery of LDOX to a tumor site involves triggering the release of the drug from its liposomal formulation in response to local heating [25,26,27]. In this case, moderate heating (≤42 °C) is preferred since temperatures greater than 43 °C are likely to cause tumor perfusion shutdown and damage the surrounding tissues [28].

Inductive moderate hyperthermia (IMH), achieved through electromagnetic irradiation, is one practical method for heat generation. In contrast to other hyperthermia methods, such as capacitive radiofrequency heating, IMH arises from both the electric and magnetic components of the applied field. We note that the magnetic component of a radiofrequency electromagnetic field penetrates through the human body with considerably lower attenuation than the electric component [29]. Electromagnetic fields provide local heating as well as influence the motion of charged particles and polar molecules in biological media [30]. For example, the application of pulsed electromagnetic fields stimulated the mineralization of Saos-2 cells [31], and this has a role in the migration and invasiveness of OS cells [32]. Importantly, the non-thermal effects of radiofrequency electromagnetic fields on ROS generation in cancer cells [33] indicate a sound rationale for combining chemotherapy with IMH to improve the efficacy of free DOX in cancer patients [34,35]. Also, the combination of regional hyperthermia with chemotherapy regimens based on free DOX in a neoadjuvant setting resulted in a higher rate of limb preservation in patients with sarcoma [36].

Previous studies have shown that various DOX nanoformulations caused a more pronounced antitumor effect in vitro and in vivo when combined with hyperthermia [37,38,39]. Comparisons between free DOX and LDOX given in combination with different hyperthermia methods (water bath and infrared heating) revealed a significantly greater inhibition of tumor growth and reduced toxicity profiles in experimental sarcoma models [27,40,41,42,43]. Another aspect of LDOX delivery in response to hyperthermia is that uniform distribution of the drug in a tumor can be expected within 40 h after treatment [44].

Although LDOX has been widely studied in different cancer cell lines, there are comparatively fewer reports on its efficacy in OS cell models. Even less elucidated are the underlying mechanisms involved in the therapeutic response to LDOX combined with IMH, which is a critical consideration for further translation into sarcoma patient treatment. We, therefore, investigated the combined effect of LDOX and IMH on the Saos-2 human OS cell line.

## 2. Results

### 2.1. Cytotoxic Response and Drug Accumulation

**Figure 1:**
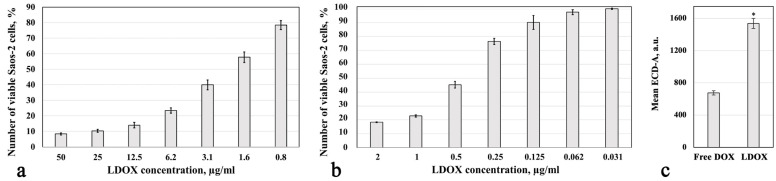
Cytotoxicity IC_50_ for LDOX in the Saos-2 cell line after 48 h (**a**) and 72 h (**b**) of incubation. Drug accumulation (**c**). *, statistically significant difference from the free DOX, *p* < 0.05.

As shown in Figure 1, prolonged exposure to LDOX significantly reduced the number of viable Saos-2 cells. There was nearly a five-fold difference in the half maximal inhibitory concentration (IC_50_) values of LDOX between 48 h (1.8 ± 0.2 μg/mL) and 72 h (0.38 ± 0.02 μg/mL) of incubation (*p* < 0.05). The levels of intracellular LDOX fluorescence in the viable Saos-2 cells are shown in Figure 1c. The liposomal formulation produced a two-point-three-fold increase in drug accumulation after 48 h of incubation compared to the free DOX (*p* < 0.05). The lower IC_50_ observed after prolonged exposure to LDOX reflected a greater accumulation of the antitumor agent in the Saos-2 cells, leading to more pronounced cell death [45,46].

The preliminary data suggested that the IC_50_ for the free DOX (0.06 ± 0.003 μg/mL) was 16% of the LDOX at 72 h, which was consistent with a previous study [47]. A comparison of the IC_50_ for the free DOX between 48 h and 72 h showed only a negligible difference. However, it should be noted that the IC_50_ value of the free DOX on the cardiomyocytes was nearly 10 times lower than that of the LDOX, which increased the risk of cardiotoxicity, as reported in [48]. Hence, in the case of LDOX, cytotoxicity in cancer cells was observed after prolonged exposure, with a potential advantage of cardio-protection [49].

**Figure 2:**
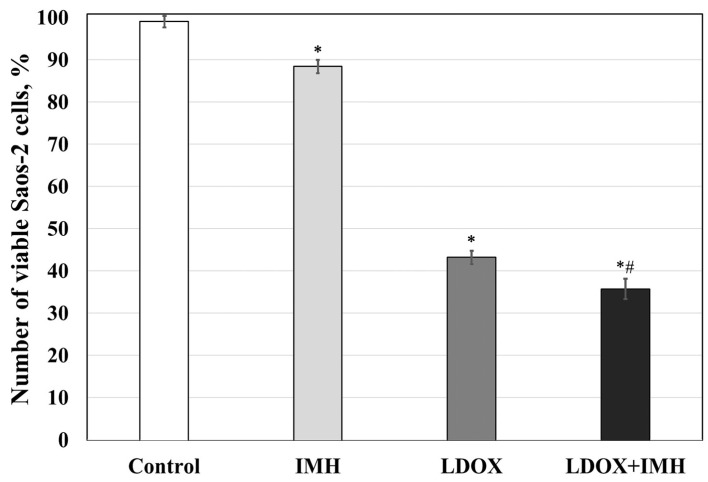
Cell viability of the Saos-2 cells exposed to the combination treatments with LDOX and IMH. *, statistically significant difference from the control, *p* < 0.05; #, statistically significant difference from the IMH treatment, *p* < 0.05.

Figure 2 shows the effect of the combination treatment with LDOX and IMH on the Saos-2 cell viability. Interestingly, IMH alone resulted in a 12% lower cell viability than the control group (*p* < 0.05). There were 57% and 61% decreases in the numbers of viable Saos-2 cells after the LDOX and LDOX plus IMH treatments, respectively, compared with the control (*p* < 0.05).

### 2.2. Apoptosis and Necrosis Detection

**Figure 3:**
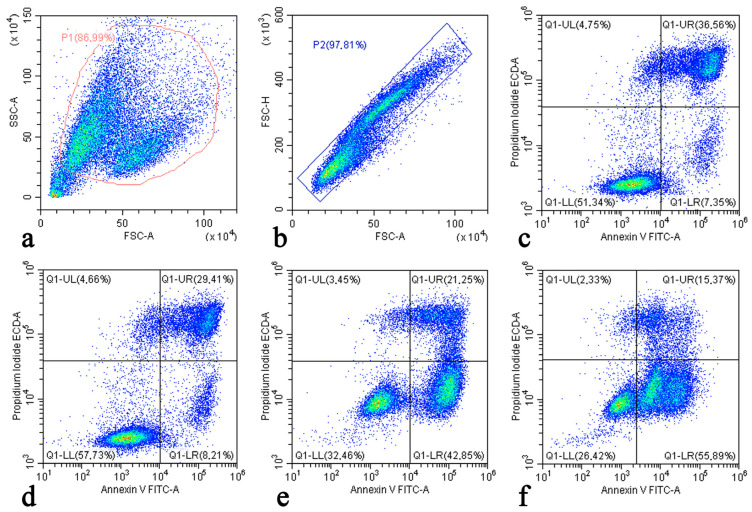
Flow cytometric studies (dot plots) of Saos-2 cells: P1 gate discarded to remove cellular debris (**a**); P2 gate with the exclusion of the doublets (**b**); FITC-A/ECD-A plot with the control cells (**c**); FITC-A/ECD-A plot with the cells after IMH treatment (**d**); FITC-A/ECD-A plot with the cells after LDOX treatment (**e**); and FITC-A/ECD-A plot with the cells after LDOX plus IMH treatment (**f**).

Figure 3 demonstrates the typical dot plots for the Saos-2 cells acquired from annexin V and propidium iodide flow cytometry studies. The cells were gated such that the cellular debris on the forward scatter area (FSC-A)/ side scatter area (SSC-A) dot plot (Figure 3a) and the cell doublets based on the FSC-A/ forward scatter height (FSC-H) plot (Figure 3b) were excluded. An analysis of the apoptosis and necrosis parameters was then performed using a four-quadrant gate on the fluorescein isothiocyanate area (FITC-A)/ energy-coupled dye area (ECD-A) plot in accordance with the annexin V and propidium iodide staining properties (Figure 3c–f).

**Figure 4:**
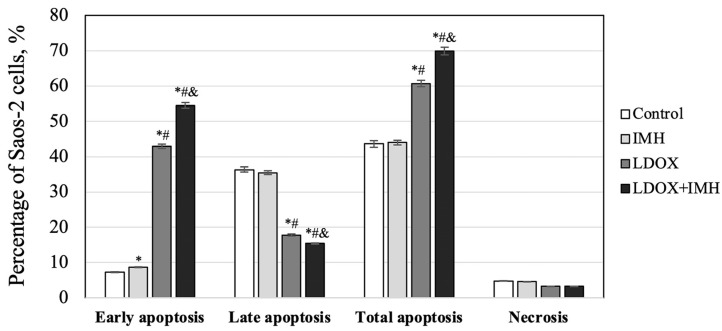
Flow cytometric analysis of apoptosis and necrosis in the Saos-2 cells. *, statistically significant difference from the control, *p* < 0.05; #, statistically significant difference from the IMH treatment, *p* < 0.05; &, statistically significant difference from the LDOX treatment, *p* < 0.05.

As shown in Figure 4, LDOX caused a 5.9 times higher number of Saos-2 cells undergoing early apoptosis in comparison to the untreated cells in the control. This confirmed that early apoptosis after LDOX treatment not only occurred in the hepatoblastoma cell line HepG2 [50] but also in the OS cell line Saos-2. In addition, there were more early apoptotic events (1.3%) detected in response to treatment with IMH alone. The Saos-2 cells exposed to LDOX in combination with IMH exhibited a 12% increase in the induction of early apoptosis compared to those exposed to LDOX alone (*p* < 0.05). Likewise, the lowest number of viable cells was evident after the combination treatment. Decreases in the populations of the Saos-2 cells in the late stage of apoptosis following the LDOX and LDOX plus IMH treatments could be associated with the ability of DOX to compete with propidium iodide for intercalary space between the base pairs of DNA molecules [51]. Overall, the LDOX plus IMH treatment led to the highest proportion of Saos-2 cells that exhibited total apoptosis (*p* < 0.05). There were no apparent differences in the percentages of the cells undergoing necrosis between the experiments.

### 2.3. Reactive Oxygen Species Measurements

**Figure 5:**
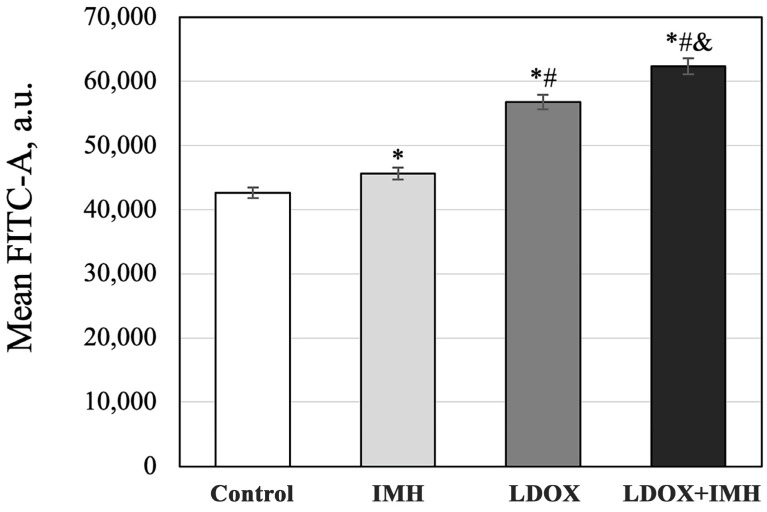
Flow cytometric analysis of ROS production levels in the Saos-2 cells. *, statistically significant difference from the control, *p* < 0.05; #, statistically significant difference from the IMH treatment, *p* < 0.05; &, statistically significant difference from the LDOX treatment, *p* < 0.05.

As measured by flow cytometry, the Saos-2 cells had elevated production levels of ROS after the IMH, LDOX, and LDOX plus IMH treatments (Figure 5). While IMH caused only a 7% increase, LDOX resulted in a 33% higher level of ROS relative to the untreated cells in the control. The ROS levels were highest in the cells treated with the LDOX and IMH combination, which was apparent as a one-point-five-fold increase in the FITC signal compared with the Saos-2 cells in the control group (*p* < 0.05).

### 2.4. Bax Expression

**Figure 6:**
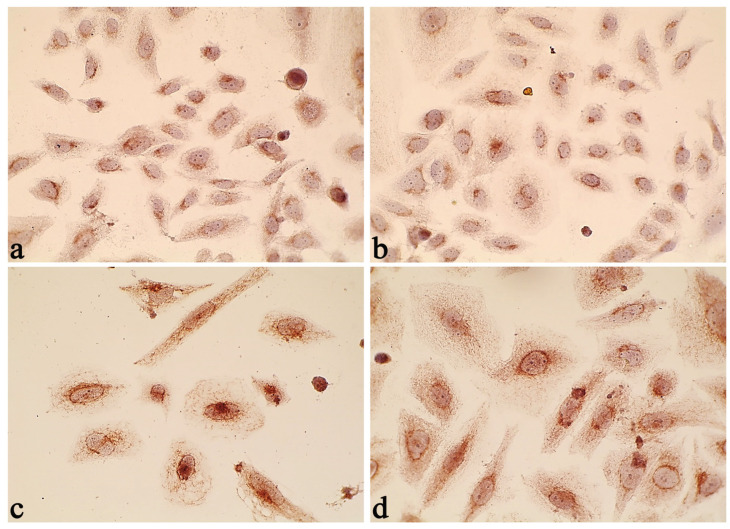
BAX expression in the Saos-2 cells: control (**a**), IMH (**b**), LDOX (**c**), and LDOX plus IMH (**d**).

**Figure 7:**
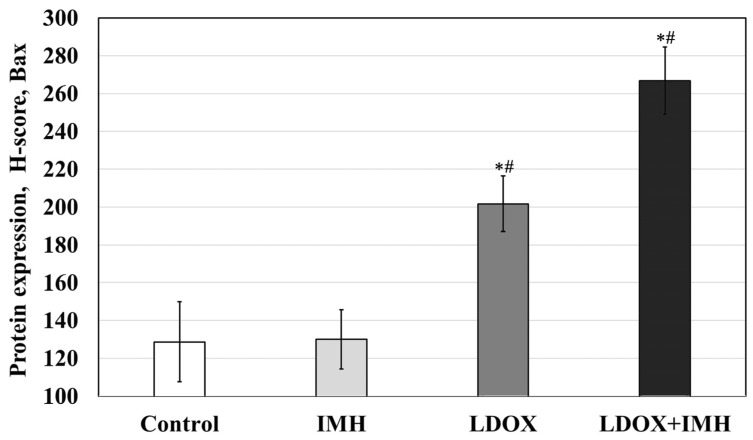
Bax expression levels in the Saos-2 cells. *, statistically significant difference from the control, *p* < 0.05; #, statistically significant difference from the IMH treatment, *p* < 0.05.

To investigate the molecular mechanisms that govern the susceptibility of Saos-2 cells to LDOX-induced apoptosis in response to IMH, we examined the pro-apoptotic Bax protein expression levels (Figure 6 and Figure 7). This protein is involved in the regulation of chemotherapy resistance [52]. The LDOX and LDOX plus IMH treatments caused 57% and 107% increases in Bax expression, respectively, compared with the untreated Saos-2 cells in the control group (*p* < 0.05). However, there was no significant difference in the Bax level between the control and the IMH treatment.

Table 1 summarizes the results of the heterogeneity analysis of the Bax distribution in the Saos-2 cells based on the spatial autocorrelation index (i.e., the Moran’s I). The LDOX plus IMH treatment showed the lowest degree of spatial heterogeneity among the LDOX, IMH, and control groups, as measured by the highest value of the Moran’s I (*p* < 0.05). Nevertheless, IMH alone led to an 11% higher Moran’s I when compared to the control group.

## 3. Discussion

In this study, we assessed the antitumor effect of a combination treatment with LDOX and IMH on Saos-2 cells. We focused our efforts on the Saos-2 cell line since it is an extensively used in vitro model of human OS with osteoblastic features [53]. Among the OS subtypes, the conventional form, histologically subdivided into osteoblastic, chondroblastic, and fibroblastic variants, remains the most prevalent [2].

**Figure 8:**
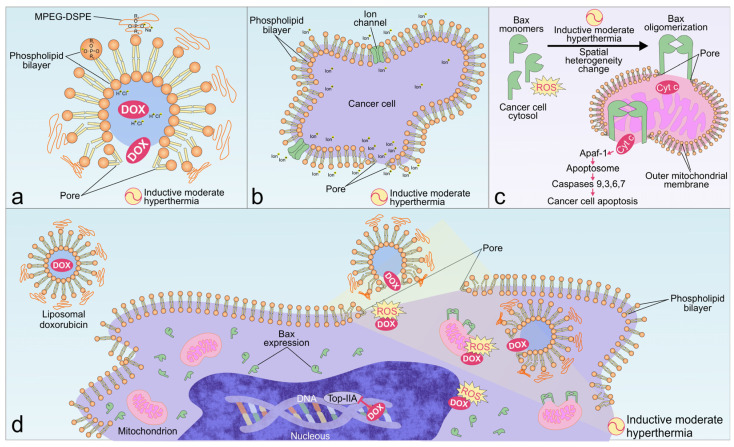
Schematic illustration of the proposed mechanism of the LDOX plus IMH action in cancer cells. (**a**) LDOX consists of an aqueous core with DOX hydrochloride enclosed within a phospholipid bilayer coated with MPEG-DSPE. IMH triggers the release of DOX by reorganization of the phospholipid bilayer and pore formation due to changes in the charge distribution directed by the electromagnetic fields. (**b**) IMH affects electron and ion transport across cell membranes through electroporation, electromechanical deformation, and moderate temperature increases, resulting in pore formation and increased DOX uptake. (**c**) Redistribution of the charges and ROS generation driven by IMH changes the spatial heterogeneity of the pro-apoptotic Bax protein in the cells, facilitating oligomerization and pore formation in the outer mitochondrial membrane. If Cyt c was released to the cell cytosol, its interaction with Apaf-1 could activate caspases 9, 3, 6, and 7 to initiate apoptosis. (**d**) IMH improves LDOX delivery through the enhanced permeability of the phospholipid bilayers driven by the induction of the eddy currents and moderate temperature rise. Once delivered to the cancer cells, DOX causes damage by ROS production, DNA intercalation, and Top-IIA inhibition, while IMH further increases ROS levels and changes the spatial heterogeneity of Bax, leading to apoptosis.

Our data confirmed previous findings [45,46] where the longer the exposure of the OS cells to LDOX, the greater the cytotoxicity (Figure 1). LDOX consists of an aqueous core with DOX hydrochloride enclosed within a phospholipid bilayer coated with N-2,2-distearoyl-sn-glycerol-3-phosphoethanolamine sodium salt (MPEG-DSPE) that helps stabilize the drug in biological media and promote its accumulation in a tumor [54]. The lowest proportion of viable Saos-2 cells found in response to the combination treatment (Figure 2) could be explained by the induced eddy currents and moderate temperature increase (<42 °C) during the IMH treatment. Our working hypothesis for the proposed mechanism of the LDOX plus IMH action in the Saos-2 cells was based on the changes in the charge distribution across the liposomal membrane, guided by IMH, that led to the reorganization of the phospholipid bilayer, enabling pore formation through which DOX could then be released (Figure 8a). At the same time, the cell membrane itself could become more permeable under the influence of IMH through similar mechanisms of electroporation and electrophoresis, thereby contributing to the cellular uptake of the drug (Figure 8b).

A number of factors come together to change membrane permeability when applying IMH, and they include ion transport, ROS generation, electromechanical deformation, and temperature effects [55,56,57]. The confirmation of the increased ROS production levels by the Saos-2 cells in response to IMH can be found in Figure 5. IMH inflicts damage by disrupting cellular structures and altering protein function, which are involved in cancer cell apoptosis and necrosis. The obtained results were in line with prior works in which other methods of hyperthermia facilitated DOX release from its liposomal formulation and enhanced cell permeability. In the same manner, the combination treatment demonstrated the efficacy of the drug delivery and the uptake of LDOX [58,59].

A greater understanding of the cell death mechanisms at early stages underlying the action of a given treatment is important for the evaluation of tumor response and toxicity [60]. For this reason, our interest in the current work centered around the early stage of apoptosis as a response to the combination treatment with LDOX and IMH. It was nevertheless observed that a larger fraction of the Saos-2 cells underwent total apoptosis after the LDOX plus IMH treatment than treatment with LDOX alone (Figure 4). As shown in Figure 5, the highest levels of ROS were measured in the cells exposed to the LDOX plus IMH treatment (*p* < 0.05).

While excessive ROS formation damages cells, low levels of ROS activate numerous signaling pathways in both normal and OS cells. For instance, ROS are well-known to play a critical role in signaling events that initiate apoptosis. The fact that electromagnetic fields modulate ROS levels could point to the potential use of IMH, when locally applied to a tumor, to increase antitumor activity and decrease the toxicity of DOX in other tissues [33,61,62]. Programmed cell death poses a barrier that restricts cancer cell survival and dissemination, wherein early apoptosis facilitates phagocytosis by macrophages without releasing pro-inflammatory cellular components, as opposed to necrosis [63,64]. Another route by which the combination treatment can affect pro-apoptotic signaling in cancer cells is via heat-shock proteins [65].

Several studies [66] have reported that the Bax protein is primarily distributed in cell cytosol as a soluble monomer under normal conditions. However, the protein undergoes a conformational shift, subsequently translocating to the mitochondria during the early stages of apoptosis. Activated Bax oligomers can further assemble into a complex that permeabilizes the outer mitochondrial membrane, facilitating the release of cytochrome-c (Cyt c). If Cyt c is released through the pores into the cell cytosol, it could interact with apoptotic protease activating factor 1 (Apaf-1) and recruit caspases 9, 3, 6, and 7 to induce apoptosis [67].

Since IMH produces both thermal and nonthermal effects, it is the combined action of heat, oxidative, and mechanical stresses that alter the regulation of protein expression [68,69]. Variations in protein expression exhibit stochastic and deterministic behaviors through a variety of dysregulated cellular pathways in cancer cells. A change in the spatial heterogeneity of Bax expression arises from cell signaling, including ROS-dependent pathways, transcriptional bursting, metabolic oscillations, and temperature perturbations which can collectively describe a larger degree of probability for apoptosis initiation via the mitochondrial pathway [70,71,72,73]. We thus cannot exclude the possibility that the lower degree of spatial heterogeneity for Bax expression after the LDOX plus IMH treatment (Table 1) was affected by a change in the protein conformational heterogeneity, which itself is a crucial step in Bax activation and, hence, apoptosis [74] (Figure 8c). More experimental work is still needed to understand some other aspects of the mechanism underlying IMH interaction with OS cells.

There is a growing body of evidence supporting considerable improvements in LDOX delivery and antitumor effects in response to hyperthermia [27,40,41,42,43]. However, to our knowledge, the significance of this work is that we examined the effect of a combination treatment with LDOX and IMH induced by a radiofrequency electromagnetic field, the magnetic component of which propagated through the biological media with lower distortion relative to its electric component, in Saos-2 cells for the first time. This was supported by [75], where the magnetic component of a single-turn loop applicator, similar to the applicator we used during IMH, propagated through human tissues with reduced distortion compared to the electric component. The present study supports the role of IMH in improving LDOX delivery and antitumor effects in Saos-2 cells (Figure 8d). The first was achieved through changes in the permeability of the phospholipid bilayers driven by the induced eddy currents and moderate temperature increase. The second relied on ROS modulation by IMH that, in principle, was involved in the activation of Bax and Saos-2 cell apoptosis [76,77].

Due to the vast heterogeneity of OS molecular profiles among patients [78], future drug delivery approaches may increasingly focus on applying IMH to trigger the release of chemotherapeutic agents from their liposomal formulations and, at the same time, enhance ROS formation. Further translation of our findings into in vivo and pilot clinical studies evaluating the efficacy and toxicity of combination treatments with LDOX and IMH is necessary to draw more definitive conclusions for sarcoma patients. Research on IMH combinations with other antitumor drugs that act on cancer cells through ROS generation and oxidative stress should be useful.

## 4. Materials and Methods

### 4.1. Cell Culture

The Saos-2 (ATCC HTB-85) OS cell line was provided by the bank of cell lines from human and animal tissue of the R.E. Kavetsky Institute of Experimental Pathology, Oncology and Radiobiology of the National Academy of Sciences of Ukraine. This cell line is commonly used in cancer research as a model of primary bone tumors characterized by osteoblastic features, alkaline phosphatase production, and DOX resistance [79,80,81]. The Saos-2 cells were cultured in Dulbecco’s modified eagle medium as follows: the nutrient mixture F12 (DMEM/F12) (Sigma-Aldrich, Taufkirchen, Germany) was supplemented with 10% fetal bovine serum (FBS) (Sigma-Aldrich, Germany). The cells were grown with an antibiotic-antimycotic (Sigma-Aldrich, St. Louis, MO, USA) at 37 °C and 5% CO_2_ [82].

### 4.2. Inductive Moderate Hyperthermia

**Figure 9:**
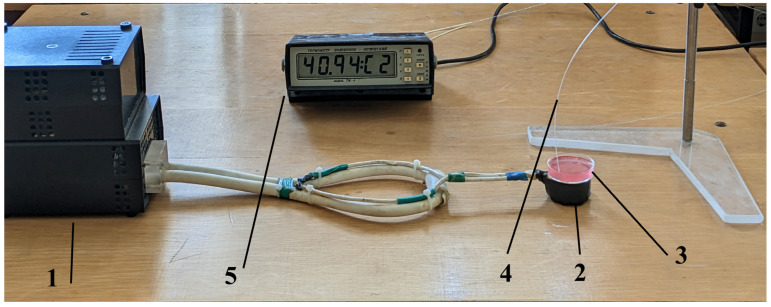
Experimental setup for the in vitro assessment of the combination treatment with LDOX and IMH in the Saos-2 cell line: the MagTherm device (1) equipped with an applicator composed of a loop and ferromagnetic dipoles (2) [83], a Petri dish with the Saos-2 cells and medium (3), a fiber optic sensor (4), and a digital thermometer (5).

As shown in Figure 9, the source of the electromagnetic irradiation used throughout the experiments was a MagTherm device (Radmir, Kharkiv, Ukraine) (1) equipped with an applicator composed of a loop and ferromagnetic dipoles (NCI, Kyiv, Ukraine) for the cell culture treatments (2) in Petri dishes (3) and a fiber optic sensor (4) wired to a TM-4 digital thermometer (Radmir, Ukraine) (5) for temperature control. The cells were exposed to electromagnetic irradiation at a 42 MHz frequency and 15 W of power for 30 min.

**Figure 10:**
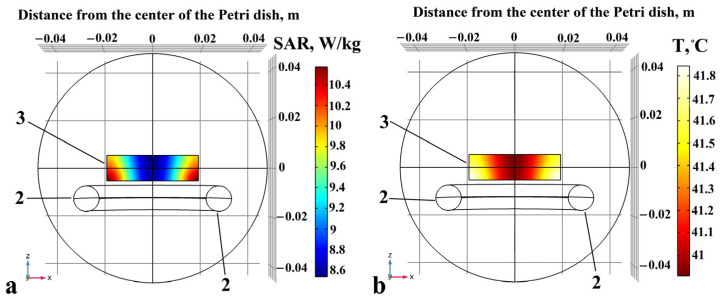
Computer-assisted planning of the SAR (**a**) and temperature (**b**) distributions produced by IMH in the cancer cells. The applicator (2) and Petri dish with the cells and the medium (3).

To produce the optimal treatment plans for the electromagnetic field and temperature distribution during the IMH treatment in the Saos-2 cells, we used COMSOL Multiphysics^®^ v. 5.6. (COMSOL AB, Stockholm, Sweden) by coupling the magnetic fields and bioheat transfer modules in the electromagnetic heating multiphysics interface. This software enabled a one-way simulation with reduced computational time and an acceptable level of model accuracy, as shown in Figure 10. The input values for the density, heat capacity, and thermal and electrical conductivity, as well as relative permittivity of the cells cultivated in the medium (Table 2), were taken from [84,85,86,87]. We adopted the frequency-transient model to compute the temperature changes over time, together with the electromagnetic field distribution in the frequency domain. The initial values of the magnetic vector potential were taken as 0 Wb/m (default values, no field). The magnetic insulation condition was assigned to the outer boundary of the model’s geometry, which set the tangential components of the magnetic potential to zero at the boundary n × A = 0. Given that the medium contained over 52 compounds, including amino acids, vitamins, inorganic salts, and fatty acids designed to mimic the cellular environment found in the human body [88], the bioheat transfer module was used to simulate thermal effects in a target biological object composed of cancer cells and medium. The initial values for the reference temperature and the initial temperature of the object were set to 37 °C. For the thermal insulation boundary conditions, the outer border of the target object assumed no heat flux across the boundary.

**Figure 11:**
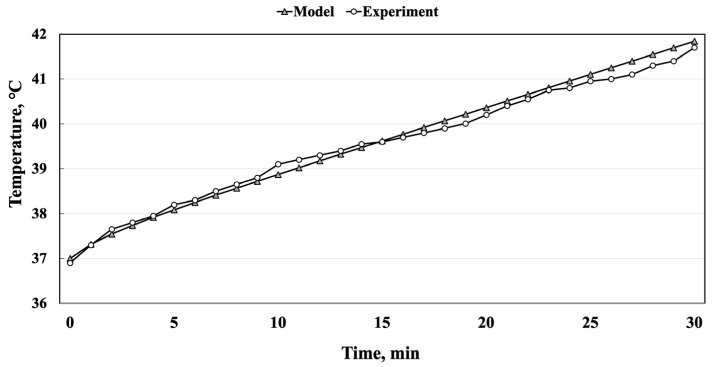
Comparison between the simulation and the experimentally measured values of the temperature over the 30 min of exposure of the Saos-2 cells to IMH (R^2^ = 0.997).

Figure 10 illustrates the expected distribution of the specific absorption rate (SAR) and temperature in a Petri dish with the Saos-2 cells in the medium after 30 min of exposure to IMH. COMSOL simulations provided a numerical basis for the IMH planning, reporting the maximum SAR value of 10.6 W/kg. The maximum magnetic field induction (B) value was 500 µT and the maximum electric field strength (E) value was 564 V/m. Also, the temperature did not exceed 41.85 °C at the edges of the dish with the Saos-2 cells, and it was 41.23 °C in its center. As shown in Figure 11, the temperatures were calibrated with the fiber optic measurements and simulations.

### 4.3. Cell Viability Assays

First, we examined the exposure time dependence of the cytotoxic response to LDOX (Dopolo, Dr. Reddy’s Natco Pharma LTD; Ministry of Health of Ukraine approval registry: L01DB01; doxorubicin catalog number: D1515, 98.0–102.0% purity determined by high-performance liquid chromatography). The Saos-2 cells were seeded at a cell concentration of 1 × 10^4^ per well in a 96-well plate (TPP, Trasadingen, Switzerland) with DMEM/F12 supplemented with FBS and an antibiotic-antimycotic (Sigma-Aldrich, USA). After adding the different concentrations of LDOX, the plates were incubated at 37 °C and 5% CO_2_ humidity for 48 or 72 h. Then, the cells were fixed with 10% trichloroacetic acid (Sigma-Aldrich, Germany), stained by sulforhodamine B dye (Sigma-Aldrich, USA), and washed with 1% acetic acid (Sigma-Aldrich, Germany). The dye elution was accomplished with a 10 mM Tris base solution (Sigma-Aldrich, Germany) [89]. The plate absorbance was read using a multi-well spectrophotometer (ThermoLabsystems Multiskan EX, Waltham, MA, USA) at 510 nm. The IC_50_ was estimated based on a nonlinear regression analysis.

Second, we determined the viability of the Saos-2 cells using the trypan blue exclusion assay (Sigma-Aldrich, Germany) in no treatment (control) and in response to IMH, LDOX, and the LDOX plus IMH combination [90]. The Petri dishes were plated with 1.5 × 10^5^ cells per dish and DMEM/F12 supplemented with 10% FBS and 1× antibiotic-antimycotic. The cells were treated with 0.4 μg/mL LDOX, followed by immediate exposure to IMH for 30 min. Subsequent to the IMH treatment, the cells were incubated under standard conditions for 72 h. Finally, the number of trypan blue-stained (nonviable) and unstained (viable) Saos-2 cells was counted in a hemacytometer (Micromed, Kyiv, Ukraine).

### 4.4. Flow Cytometric Assessment of Drug Accumulation

The Saos-2 cells exposed to LDOX or free DOX based on the IC_50_ values and incubated under standard conditions for 48 h were analyzed for their cellular fluorescence in a DxFlex flow cytometer (Beckman Coulter, Brea, CA, USA) using the CytExpert v.2.5 software. The fluorescence intensity was measured from the ECD channel [91]. The gating strategy used FSC-A and SSC-A to detect viable, single-cell events. A total of at least 25,000 events were collected from each sample.

### 4.5. Flow Cytometric Assessment of Apoptosis and Necrosis

The population of Saos-2 cells undergoing apoptosis was detected by flow cytometry using staining with annexin V FITC and propidium iodide ECD (Dojindo, Munich, Germany) [92]. The cells were either given no treatment (control) or exposed to the IMH, LDOX, or LDOX plus IMH combination treatments as described above and subsequently incubated for 48 h under standard conditions. Then, the cells were stained with annexin V FITC and propidium iodide ECD according to the manufacturer’s instructions, incubated for 1 min, and analyzed on a DxFlex flow cytometer (Beckman Coulter). A total of at least 21,000 events were acquired for each sample and analyzed using CytExpert v.2.5 software for DxFlex.

### 4.6. Flow Cytometric Detection of Reactive Oxygen Species

The ROS levels were measured using a fluorescent probe 2’,7’-dichlorodihydrofluorescein diacetate (DCFH-DA, Dojindo, Germany) as in [93]. Gating was performed to separate the Saos-2 cells, and the data were analyzed as above.

### 4.7. Immunocytochemical Assay

The Saos-2 cells were incubated for 72 h under standard conditions in the control (no treatment) and the IMH, LDOX, or LDOX plus IMH groups and then fixed with a methanol solution (Sigma-Aldrich, USA) and acetone (Chimreserv, Kyiv, Ukraine). The cells were subsequently probed with primary antibodies to Bax (clone 6A7, Thermo Scientific, Waltham, MA, USA), which recognize an epitope of the activated protein form [94,95], and incubated at room temperature for 1 h. An immunocytochemistry assay provided an integral visualization of the Bax expression in the cells. Immunolabeling was detected using a SuperPicture Polymer Detection Kit (Thermo Fisher Scientific, USA) with a hematoxylin counterstain for 2 min. Bax protein expression was assessed by an H-score [96].

### 4.8. Image Analysis

First, the Saos-2 cells were segmented from the acquired immunocytochemistry images based on k-means clustering using ImageJ v.1.53k (NIH, Bethesda, MD, USA) software. We then calculated the Moran’s spatial autocorrelation index as a quantitative measure of heterogeneity in the intracellular Bax distribution with Autocorrelation v.1.0. (NCI, Kyiv, Ukraine) [97,98].

### 4.9. Statistical Analyses

The statistical analyses were performed with Statistica 6.0 (Statsoft Inc., Tulsa, OK USA) and IBM SPSS Statistics 25.0 (IBM Inc., Chicago, IL, USA). The Kolmogorov–Smirnov and Shapiro–Wilk tests were used to assess whether the data were normally distributed. Differences among the groups were analyzed by Student’s t-tests, one-way ANOVA followed by Games–Howell post hoc tests, or Kruskal–Wallis tests. Statistical significance was accepted at *p* < 0.05.

## 5. Conclusions

In summary, this study demonstrated the effects of a combination treatment with LDOX and IMH on Saos-2 cells. As a single agent, the IC_50_ value of LDOX was five times lower in response to prolonged exposure at 72 h than it was at 48 h (*p* < 0.05), which was attributed to a greater accumulation of the drug in the Saos-2 cells. This result was in agreement with an earlier study on employing liposomal formulations to achieve the improved targeting and therapeutic efficacy of DOX [99]. IMH planning of the radiofrequency electromagnetic field distribution, the magnetic component of which propagated through the biological media with lower distortion relative to its electric component, showed that the temperature did not rise above 41.9 °C in a Petri dish with the cells and medium. The LDOX and IMH treatment resulted in a 61% reduction in the Saos-2 cell viability compared with the control group. There was a 12% increase in the population of cells undergoing early apoptosis after the combination treatment compared to the LDOX treatment. The highest levels of ROS were found in the cells exposed to the LDOX plus IMH treatment. While LDOX alone or in combination with IMH was associated with a significantly higher level of Bax expression, the texture analysis of the immunocytochemistry images revealed that the pro-apoptotic protein was less heterogeneously distributed in the Saos-2 cells following the combination treatment than it was after a single agent use (*p* < 0.05). In line with previous studies [100], lower levels of tumor cell heterogeneity can be considered as an additional pro-apoptotic factor in the evaluation of a treatment response. Our hypothesis concerning the proposed mechanism of the LDOX plus IMH action was based on the improved delivery of the drug through changes in the permeability of the phospholipid bilayers in the liposomes and the Saos-2 cells and the enhanced antitumor effect of DOX initiated by increasing ROS production, reducing the level of visualized Bax heterogeneity in the cells and inducing apoptosis. Furthermore, this provides a rationale for translating the obtained results into in vivo studies evaluating the efficacy and toxicity of combination treatments with LDOX and IMH in animal models of OS.

## Figures and Tables

**Table 1:** Spatial heterogeneity analysis of the BAX distribution in the Saos-2 cells.

Experiment	Moran’s I, a.u.
Control	0.40 ± 0.004
IMH	0.45 ± 0.004 *
LDOX	0.53 ± 0.008 *^#^
LDOX plus IMH	0.58 ± 0.005 *^#&^

*, statistically significant difference from the control, *p* < 0.05; ^#,^ statistically significant difference from the IMH treatment, *p* < 0.05; ^&^, statistically significant difference from the LDOX treatment, *p* < 0.05.

**Table 2:** Physical parameters of the cancer cells cultivated in the medium.

Object	Parameter	Value
Medium	Density	1018 (kg/m^3^)
	Heat capacity at constant pressure	3930 (J/(kg*K))
	Thermal conductivity	0.496 (W/(m*K))
	Electrical conductivity	1.24 (S/m)
	Relative permittivity	1.0
Cancer cells	Density	1090 (kg/m^3^)
	Heat capacity at constant pressure	3900 (J/(kg*K))
	Thermal conductivity	0.49 (W/(m*K))
	Electrical conductivity	1.5 (S/m)
	Relative permittivity	55.0

## Data Availability

The data presented in this study are available on request from the corresponding author.
